# New PPARG Exons: Cell-Specific Expression of Their RNAs in the Human Placenta

**DOI:** 10.3390/cells15070639

**Published:** 2026-04-01

**Authors:** Marie-Léone Vignaud, Nathalie Morin, Thierry Fournier

**Affiliations:** Université Paris Cité, Inserm, UMR-S1139, FPRM, F-75006 Paris, France; nathalie.morin@u-paris.fr (N.M.); thierry.fournier@u-paris.fr (T.F.)

**Keywords:** *PPARG* gene, 5′UTR exon, trophoblast differentiation, cell-type-specific expression, placenta, pregnancy

## Abstract

**Highlights:**

- The *PPARG* gene comprises fourteen exons, ten previously described and four new ones.
- Cell-type-specific and placental environment-specific expression of *PPARG* transcripts in the human placenta.

**What are the main findings?**

- Two new exons are specifically expressed in villous cytotrophoblasts and may be involved in oxygen adaptation during the first trimester of pregnancy.

**Abstract:**

Peroxisome proliferator-activated receptor γ (PPARγ), encoded by the *PPARG* gene on chromosome 3p25.2 in humans, is a ligand-dependent transcription factor that belongs to the nuclear receptor family. In various tissues, PPARγ controls cell differentiation, proliferation, or fusion. Its essential role in the development and functions of the placenta is now well established. To date, the specific functions of its RNA isoforms, encoded by ten exons, in trophoblast biology, including cell fusion and invasion, remain unknown. As translation is mainly regulated by the 5′UTR sequences of mature mRNA, this region was analyzed, and four previously unreported exonic sequences were revealed. Their expressions were confirmed and quantified in villous cytotrophoblasts from term placenta and in chorionic villi from both first-trimester and term placenta. Distinct expression patterns were observed: one exon showed weak expression in placental and chorionic cells, another exhibited stable expression throughout pregnancy, while two exons specific to villous cytotrophoblasts displayed increased expression during the first trimester, suggesting a role in oxygen-responsive mechanisms. Among these, one may be involved in villous trophoblast differentiation. These findings demonstrate that the *PPARG* gene is composed of 14 exons and is highly regulated depending on cell type and the stage of cell differentiation.

## 1. Introduction

Human placenta is a transitory endocrine organ essential for implantation, fetal development and growth, and maintenance of pregnancy. It contributes to the immune tolerance of the conceptus and participates in the remodeling of uteroplacental vascularization. The placental chorionic villous represents the structural and functional unit of the human placenta and is also a selective biological barrier between maternal and fetal blood. The chorionic villi are composed of different cell types. They consist of fetal capillaries within a mesenchymal core also composed of fibroblasts and macrophages (Hofbauer cells). This axis is bordered by the villous cytotrophoblast (VCT). The VCTs form a layer of mononuclear epithelial cells whose function is to fuse with the overlying syncytiotrophoblast (ST) in contact with the maternal blood to constantly renew the syncytium throughout pregnancy. This ST enables the exchanges of gases and nutrients necessary for embryo development and fetal growth. It is also an endocrine tissue that produces several hormones (human chorionic gonadotropin and steroids). At the tip of the chorionic villi anchored in the decidua basalis, on the maternal side, extravillous cytotrophoblasts (EVTs) proliferate and invade the decidua, reaching the upper third of the myometrium in the first trimester of pregnancy. Then, the EVTs undergo an epithelial to mesenchymal transition (EMT) to acquire a migratory and invasive phenotype, specifically toward the maternal spiral decidua arteries, and participate in their remodeling into atonic vessels. EVTs are also involved in the immunotolerance of the semi-allogenic graft represented by the conceptus by interacting with maternal immune cells (natural killer cells, T lymphocytes, macrophages) [1].

The peroxisome proliferator-activated receptor γ (PPARγ) is a ligand-dependent nuclear receptor that functions as a transcription factor. It is expressed in other tissues such as intestine, muscle, and placenta. It forms an exclusive heterodimer with the 9-cis retinoic acid receptor that is essential for its binding to DNA at the consensus sequence in the promoter of target genes and activates transcription. As a transcription factor, PPARγ is involved in the expression of a large number of genes that control metabolism and particularly lipid metabolism, inflammation, cell proliferation and differentiation, response to oxidative stress, and apoptosis [2,3,4,5].

PPARγ functions are well established in mice and human placental development [6,7,8,9]. In humans, PPARγ controls both villous and EVT differentiation in vitro [1,10,11]. In vivo, *PPARG* polymorphisms are associated with an increased risk of pathological pregnancies such as preeclampsia. Mutations in the ligand-binding domain of PPARγ are responsible for familial partial lipodystrophy type 3, which is characterized by severe metabolic complications such as hypertension, insulin resistance, lipoatrophy, and complications of pregnancy when the placenta carries the mutation [12,13,14].

However, the regulation of *PPARG* gene expression is largely unknown. The *PPARG* gene, located on chromosome 3p25.2 in humans, is more than 150 kb long. To date, it is thought to contain ten or eleven exons, including four or five in the 5′UTR genomic region labeled A1.1, A1.2, A2, and B or A1, A2, B, C, and D, but the structure of the 5′UTR region remains incompletely described [15,16]. The other six exons make up the gene’s coding sequence and the 3′UTR sequence [17]. In order to understand the regulation of mature PPARG mRNA translation in human placenta and to clarify the 5′UTR region, we analyzed the 5′UTR sequences of the 16 transcripts coding for eight PPARγ isoforms included in the NCBI database. The data analysis revealed four sequences that had not previously been described as exons. We confirmed and quantified their expression in human placenta obtained from uncomplicated pregnancies. Our results suggest their involvement in trophoblast differentiation and adaptation to oxygen in placenta. Moreover, the expression of two of them seems to be villous trophoblast-specific.

## 2. Materials and Methods

### 2.1. Ethical Statement

This study complies with the Declaration of Helsinki and was approved by the local ethics committee (CPP 2015-mai-13909). The placentas used in this study were obtained after receiving informed consent from patients who underwent voluntary termination of pregnancy or scheduled cesarean section. The samples came from normal full-term pregnancies (between 38 and 41 weeks of amenorrhea) or voluntary terminations of pregnancy between 6 and 13 weeks of amenorrhea in patients who reported being non-smokers or very light smokers (Table 1). All placentas used came from the maternity wards of Hôpital Cochin—Port Royal, Hôpital des Diaconesses, Hôpital privé d’Antony, and Institut Mutualiste Montsouris.

### 2.2. In Silico Analysis

In silico analysis of PPARG transcripts referenced on NCBI in March 2022 (https://www.ncbi.nlm.nih.gov/datasets/gene/id/5468/products/, accessed 1 March 2026) was performed with BioEdit (version7.5.2) and Blastn software (https://blast.ncbi.nlm.nih.gov/Blast.cgi, accessed 1 March 2026). The human *PPARG* gene NC_000003.12 (12287368…12434344) was used as the reference sequence. Briefly, the 5′UTR of each transcript included in NCBI database was downloaded, and BLAST analysis was conducted on the human *PPARG* gene using Blastn software. The locations, on the gene, of each exon found are noted in Supplementary Table S1. The location of the previously described exon was established according Sundvold et al. and Omi et al. [15,18].

The ENCODE database (https://www.encodeproject.org/) was used to determine whether the potential promoters identified have high chromatin accessibility, H3K4me and/or H3K27ac signals, and are farther than 200 bp or 2 kb from an annotated transcription start site. Trimethylation of lysine 4 on histone H3 (H3K4me3) is commonly associated with transcriptionally active promoters and contributes to gene activation by promoting an accessible chromatin environment and facilitating transcription factor recruitment [19,20]. Acetylation of histone 3 at lysine 27 (H3K27ac) is widely considered as a hallmark of active enhancers and correlates with transcriptional activation [21,22].

### 2.3. Isolation of Chorionic Villi and Trophoblastic Cell Cultures

The placental tissues were recovered quickly after the surgical procedure. For term placentas, the basal and chorionic membranes were removed, and the cotyledons were rinsed three times in Hank’s balanced salt solution (HBSS) without Ca^2+^ and Mg^2+^ (Gibco^TM^, Thermo Fisher Scientific, Illkirch, France) at room temperature. The tissue was then dissected to isolate the villi in HBSS. The first-trimester placentas were obtained after voluntary termination of pregnancy performed by aspiration. The membrane was removed, and the chorionic villi were isolated in HBSS. Villi from term or first-trimester placentas were either immersed in liquid nitrogen and frozen at −80 °C pending their next use or used to isolate VCTs from term placenta.

The minced tissue (~15 g) was digested at 37 °C for 30 min in a shaking incubator (50–60 rpm) with a filtered trypsin solution containing 500 mg of trypsin powder ((Gibco #27250-018), 250 μL of 0.1 M MgSO_4_ ((Merck #5886-0500, New York, NY, USA), 250 μL of 0.1 M CaCl_2_ (Merck #1-02820-1000), 75 μL of DNase I (50 U/mL) (Sigma-Aldrich #D5025, Saint Quentin Fallavier, France), and 10 mL of fat-free milk in 250 mL of warm 1X HBSS. This process was repeated with additional digestion media (50 mL and 30 mL) for up to 4–5 cycles, maintaining the same temperature and shaking conditions. Enzymatic digestion was monitored under a microscope and stopped by filtering the mixture through a 40 μm filter in fetal calf serum (Eurobio^TM^ #CVFSVF00-01, 10% final concentration, Les Ulis, France). The resulting cell suspensions were centrifuged at 280× *g* for 10 min at room temperature. The cell pellets were then suspended in Dulbecco′s modified Eagle′s medium (DMEM, containing 1 g/L glucose, pyruvate, without phenol red; Thermo Fisher Scientific, #11880, Illkirch, France; Gibco^TM^ #11880-028) and layered on a preformed Percoll gradient (70% and 5%) (Cytiva^TM^ #17-5445-01, Uppsala, Sweden). Centrifugation at 1400× *g* for 20 min at room temperature yielded an interface layer containing the VCTs, which was collected, washed in DMEM, and centrifuged again at 1200 rpm for 10 min.

The final cell pellet was resuspended in complete DMEM supplemented with 10% FCS (Eurobio #CVFSVF00-01, Les Ulis, France), 2 mM of glutamine (Sigma-Aldrich #G7513, Saint Quentin Fallavier, France), 100 IU/mL penicillin, and 100 μg/mL streptomycin (Gibco #15140-122, Thermo Fisher Scientific) (1% GlutaMAX, Gibco^TM^ #3505-038, 10% FCS and 1% pennistreptomycin, Gibco^TM^ #115140-122) and counted using a TC20^TM^ Automated Cell Counter (Bio-Rad, Hercules, CA, USA). Cells were either snap-frozen and stored at −80 °C or seeded at a density of 125,000 cells/cm^2^ in 60 mm culture dishes and incubated overnight in a 5% CO_2_ atmosphere at 37 °C. VCTs were recovered by trypsin-EDTA 0.05% (Gibco #25300054) after 24 h, 48 h, or 72 h of culture, washed in PBS, snap-frozen and stored at −80 °C.

The BeWo cell line, used as a human VCT model, was cultured in DMEM-F12K medium (Thermo Fisher Scientific, Illkirch, France), supplemented with 10% FCS and 1% streptomycin and penicillin, and incubated overnight in a 5% CO_2_ atmosphere at 37 °C. The cells were recovered by trypsin-EDTA 0.05% (Gibco #25300054) after 24 h of culture, washed in PBS, snap-frozen, and stored at −80 °C.

### 2.4. RNA Extraction, Reverse Transcription, PCR, and Real-Time Quantitative PCR

mRNA extraction from term villi and voluntary termination villi required a preliminary step of grinding with an Ultra-Turrax homogenizer T25 (IKA, Staufen, Germany). Total RNA extraction of VCT was performed on pellets of 3 million cells with the RNeasy mini kit (Qiagen #74104, Hilden, Germany) according to the supplier’s instructions, with DNase (Qiagen #79254). Elution was performed with RNase- and DNAse-free water. RNA concentration and purity were verified using a spectrophotometer (Nanodrop ND-1000, Thermo Fisher Scientific, Milwaukee, WI, USA). RNAs were stored at −80 °C after addition of 1 µL of RNase-OUT (Invitrogen #10777019, Carlsbad, CA, USA). Reverse transcription was performed using the Transcriptase Inverse SuperScript^TM^ III kit (Invitrogen #18080044) using oligodT primer (Invitrogen # 18418012) for 1 µg of RNA according to the supplier’s instructions. Prior to performing RT-qPCR, the primers (Table 2) provided by Eurogentec were validated beforehand and found to be more than 98% effective [22]. The primers used and their characteristics are detailed in Table 1. For the HLA-G target, the transmembrane region of the protein, specific to the HLA-G1, HLA-G2, HLAG-3, and HLA-G4 isoforms, was used [23]. To perform PCR, five µL of cDNA-oligodT at a concentration of 25 ng/µL was added to 45 µL of Phusion Flash High-Fidelity PCR Master Mix (Thermo Scientific^TM^ F548L) and forward and reverse primers at a final concentration of 0.5 µM. PCR reactions were performed using a thermocycler (SimplyAmp Applied Biosystem^®^, Thermo Scientific). The amplification protocol included a preliminary denaturation step at 95 °C for 10 min, followed by 40 PCR cycles comprising a denaturation step at 95 °C for 30 s, hybridization at the most appropriate temperature for the primer pairs (see Table 2) for 30 s, and elongation at 72 °C for 1 min 30 s. The cycle ended with an elongation step at 72 °C for 10 min. An amount of 10 µL of PCR product was migrated on a 1.5% agarose gel for 1 h 30 min to 2 h at 120 V.

Placental sex was determined as described by Degrelle et al. Briefly, a multiplex PCR targeting the chromosomes X and Y using Phusion Flash High-Fidelity PCR Master Mix (Thermo Scientific^TM^ F548L) with 10 ng of cDNA per reaction from reverse transcription with oligo-dT primers was performed following the manufacturer’s instructions [24].

RT-qPCR was carried out in a 10 µL reaction volume containing 10 ng of cDNA, 5 µM of each primer, and 5 µL of SYBR^TM^ Green (Master Mix PowerTrack, Applied Biosystems #A46111, Vilnius, Lithuania) completed with RNase- and DNAse-free water using the CFX384 Real-Time PCR Detection System (Bio-Rad). PCR cycles consisted of the following steps: polymerase activation (2 min, 95 °C), denaturation (15 s, 95 °C), and annealing and extension (1 min, 60 °C), followed by a melting curve to ensure no contamination. The threshold cycle (Ct) was measured as the number of cycles at which the fluorescence emission first exceeds the background.

mRNA levels were normalized by averaging of housekeeping genes: SDHA, RPLP0. The relative amounts of mRNA were estimated using the 2^−ΔΔCt^ method for relative comparison between VCT and chorionic villi expressions or chorionic villi at term and villi from first trimester of pregnancy and then expressed as fold change with the term villi as reference group.

### 2.5. Statistics

All measurements were performed in at least three independent experiments (3 to 7). All statistical analyses were performed using GraphPad PRISM v. 10.0 (GraphPad Software, Inc., San Diego, CA, USA). All numerical data are expressed as mean ± SD. For all statistical analyses, the normality of the distribution was tested using the Shapiro–Wilk test followed by a two-way ANOVA test. Means were considered statistically different for a *p*-value < 0.05 and are shown on a graph; *: *p*-value ≤ 0.05, **: *p*-value < 0.01, ***: *p*-value < 0.001.

## 3. Results

### 3.1. In Silico Analysis of 5′UTR PPARG NCBI Transcripts Reveals Four New Exons and Four New Potential Promoters on the PPARG Gene

The NCBI database contains 16 reference transcripts for the *PPARG* gene. Their 5′UTR sequences were retrieved and analyzed. The sequences ranged in length from 85 to 647 nucleotides (Table S1 Supplementary Material). In silico analysis showed that 11 different sequences could make up this region.

Four of these have already been described and are labeled A1.1, A1.2, A2, and B by Omi et al. [15]. It should be noted that only transcript NM_001354669.2, which codes for PPARG isoform 5 (PPARG5), possesses in its 5′UTR region three exons normally described as constituting the PPARG coding sequence (exons 1 to 3) in addition to exons A1.1 and A2.

Four other sequences, not previously described, were identified and named X1, X3, X4, and X5. Each of these is present in only one transcript referenced in the NCBI database. The first three code for mRNA PPARG isoform 1 (PPARG1), and the sequences identified (X1, X3, X4) are located at the beginning of the transcript. The last sequence codes for mRNA PPARG isoform 3 (PPARG3) and X5 is located at the end of the 5′UTR region of transcript NM_001374262.3, just before the coding sequence.

**Figure 1:**
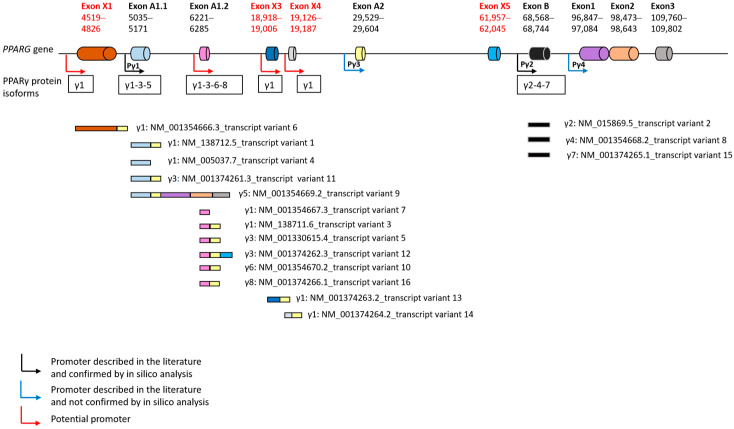
Four new exons in the schematic representation of the 5′UTR exon and intron in human *PPARG* gene and the 5′UTR of the NCBI referenced transcripts. The annotation of the exon was based on Omi et al. [15]. New exons are written in red. Promoters described in the literature are in black or blue; the location of possible promoters is in red. Black promoters (Pγ1, Pγ2) were found by in silico analysis. Blue promoters (Pγ3, Pγ4) were not found by in silico analysis. The transcription of the PPARγ protein isoform is shown in the box below each potential and described promoter.

X1, located between 4519 and 4826 bp (301 bp) on the *PPARG* gene, is found in NM_001354666.3. Two others (X3, X4), located between 18,918–19,006 and 19,126–19,187 (89 bp and 64 bp), are present in NM_001374263.2 and NM_001374264, respectively. X5 is located between 61,957 and 62,045 bp (97 bp), between exons A2 and B (Figure 1).

The data show six possible transcription sites starting before exon X1, A1.1, A1.2, X3, X4, and B. Only 4 PPARG promoters have been reported in the literature, before exon A1 (A1.1), A2, B, and exon 1 for promoters labeled Pγ1, Pγ3, Pγ2, and Pγ4, respectively [18]. The data seem to indicate that the previously described Pγ1 promoter could be used for the transcription of PPARG1, PPARG3, and PPARG5 mRNA isoforms. The Pγ2 promoter could transcribe all mRNA isoforms containing exon B (PPARG2, PPARG4 and PARRG7). It should be noted that the sequences analyzed did not reveal any transcripts starting just before exon A2 or exon 1 corresponding to the Pγ3 and Pγ4 promoters, respectively, described by Sundvolt et al. [18]. Interestingly, this analysis identified four other potential promoters (shown in red in Figure 1) upstream of exon X1, A1.2, X3, and X4 which could be used for the transcription of the PPARG1, PPARG3, PPARG6, and PPARG8 mRNA isoforms.

Chromatin accessibility, H3K4me and/or H3K27ac signals, and the presence of a nearby transcription start site are key factors in supporting the hypothesis of a promoter [20,25]. The ENCODE database was consulted to identify the chromatin signature upstream of exons X1, A1.2, X3, and X4. The data indicate that the regions likely to contain potential promoters all belong to distal enhancers characterized by high chromatin accessibility with H3K27ac signals and are located more than 2 kb from an annotated transcription start site.

### 3.2. Trophoblast-Specific Expression of X3, X4, and X5 Exons and Possible Involvement of X4 in Villous Cytotrophoblast Differentiation

**Figure 2:**
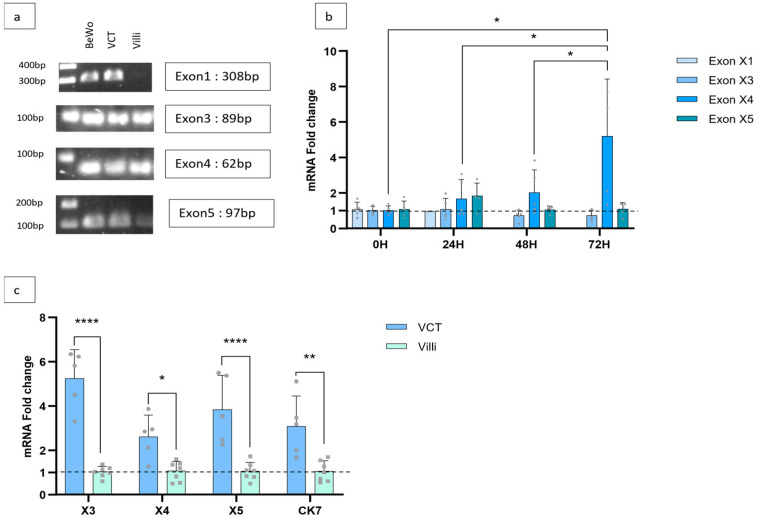
Detection and expression during VCT differentiation and specific expression of exons X3, X4, and X5 in human placental trophoblasts. (**a**) PCR targeting X1, X3, X4, and X5 in BeWo cells after 24 h of culture, VCT after isolation, and term chorionic villi. (**b**) Evolution of exon X1, X3, X4, and X5 expression during syncytiotrophoblast formation. Quantification of X1, X3, X4, and X5 carried out in cytotrophoblasts (*n* = 5) after isolation (0 H) and in in vitro culture at 24 h, 48 h, and 72 h (24 H, 48 H, 72 H). *n* = 6 *: *p*-value ≤ 0.05. 0 H was used as reference. (**c**) Comparison of the expression of exons X3, X4, X5 and CK7 between VCT cells (*n* = 5) and villi (*n* = 5). Villi were used as reference. X1 was barely detectable and not quantified. *: *p*-value ≤ 0.05, **: *p*-value <0.01, ****: *p*-value <0.0001.

Since PPARγ plays a very important role in placental development, we sought to determine whether these sequences were expressed in VCTs and chorionic villi obtained from physiological human placentas at term. PCRs targeting all the sequences of X1, X3, X4, and X5 were performed on the BeWo cell line, VCTs, and chorionic villi from non-pathological term pregnancies, and the amplicons were sequenced. The BeWo cell line originates from choriocarcinoma, a trophoblastic tumor. It is commonly used in in vitro models to study trophoblastic differentiation. It is known to express PPARγ, making it a model for validation and positive controls. Sequencing confirmed the expression of X1, X3, X4, and X5 in all samples tested (Figure 2a).

In in vitro culture, mononucleated VCTs aggregate and fuse into a polynucleated syncytiotrophoblast within 72 h [26]. Therefore, the expressions of X1, X3, X4, and X5 were quantified by RT-qPCR in VCTs, from isolation to 72 h of culture (0 h, 24 h, 48 h, and 72 h), in order to monitor changes occurring during trophoblast differentiation. X1 had a very low expression in the VCT between 0 h and 48 h of culture and was not detected at 72 h. Expression of X3, X4, and X5 was stable up to 48 h of culture. Only X4 expression significantly increased at 72 h (Figure 2b).

Chorionic villi are mainly composed of trophoblasts (EVT, VCT, and ST) and of endothelial and mesenchymal cells [27]. To determine which components of the villi express X1, X3, X4, and X5, we compared their expression between freshly purified VTCs and whole villi.

X1 expression was barely detectable in VCT and undetectable in villi. We can conclude that the transcript is not or very poorly expressed in other components of villi. The expression of the X3, X4, and X5 sequences was greater in VCT than in villi, suggesting that they are mainly expressed in the trophoblast layer rather than in the whole tissue (Figure 2c).

To confirm these results, we quantified CK7 expression, a specific marker of human trophoblasts, in the two groups [28]. CK7 expression was three times higher in VCT samples than in villi (Figure 2c). These results confirm that X3, X4, and X5 and, therefore, their transcripts are expressed specifically by the trophoblast in placental tissue.

### 3.3. Evolution of Expression of New PPARG Exons of During the First Trimester

#### 3.3.1. Placental Oxygen Levels During the First Trimester of Pregnancy Have an Impact on Expression of New X3 and X4 Exons

Early placentation is crucial for the maintenance of pregnancy and is a finely regulated mechanism. In particular, oxygen pressure in the intervillous space is a key factor in fetal implantation. Until the eighth week of pregnancy, the placenta develops in a relatively hypoxic environment, with a partial pressure of oxygen (pO_2_) below 20 mmHg. The dissolution of trophoblastic plugs obstructing the maternal uterine arteries between the 8th and 10th week of pregnancy allows oxygenated arterial blood to increase and maintain pO_2_ at 60–80 mmHg from the 10th week of pregnancy until term [29,30,31]. We decided to study the expression of X1, X3, X4, and X5 during the three phases of implantation—before 8 weeks of pregnancy when the oxygen level is low, between 8 and 10 weeks when the trophoblastic plugs break down, and between 10 and 14 weeks when oxygen pressure has increased—and to compare them with their expression at term.

**Figure 3:**
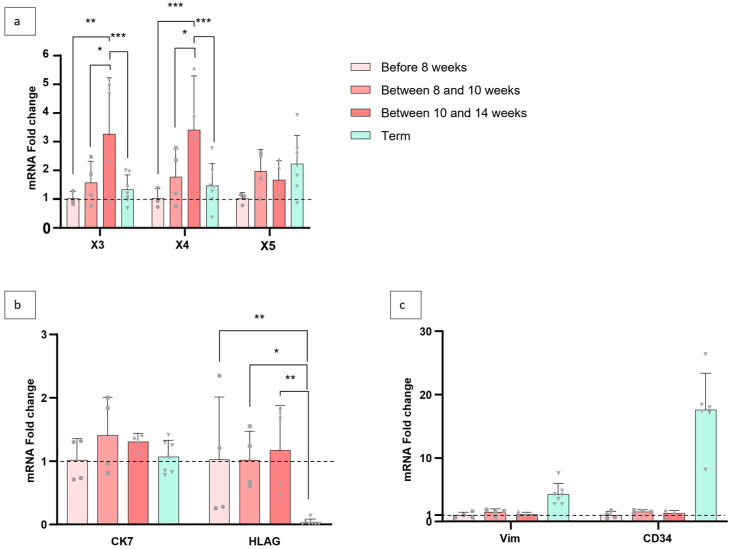
Overexpression of X3 and X4 correlates with the oxygen level in the placental environment. (**a**) Relative quantification of X1, X3, X4, and X5 expression in villi; at three stages of the first trimester corresponding to variations in oxygen levels; and at term. X1 was barely detectable, and not quantified. *: *p*-value ≤ 0.05, **: *p*-value <0.01, ***: *p*-value <0.001. *n* = 3 or 4 for each time of the first trimester, and *n* = 6 for the term. Before 8 weeks was used as reference. (**b**,**c**) Relative quantification of CK7, HLA-G, vimentin, and CD34 expressions in three stages of the first trimester and at term. *: *p*-value ≤ 0.05, **: *p*-value <0.01. *n* = 3 or 4 for each time of the first trimester, and *n* = 6 for the term. Before 8 weeks was used as reference.

X1 was barely detectable and not quantified. X5 showed no significant change in expression during the four stages. The expression of X3 and X4 was significantly upregulated during the two later stages of the first trimester (between 8 and 10 weeks and after 10 weeks). At term, the expressions of X3 and X4 were comparable to those before eight weeks. This observation suggests that transcripts containing X3 and X4 appear to be regulated by oxygen level (Figure 3a). The transcription factor hypoxia-inducible factor is known to be involved in the response to hypoxia. Its response element (hypoxia-responsive element) was not identified upstream of exons X3 and X4.

During early placentation, EVTs attach to the decidua basalis, proliferate, and undergo an epithelial–mesenchymal transition. These invasive EVTs infiltrate the maternal decidua and extend to the upper third of the myometrium. They also migrate specifically to the uterine spiral arteries and participate in their remodeling. As mentioned above, CK7 is a specific marker of human trophoblasts. It is therefore not possible to distinguish between villous and extravillous trophoblasts using this marker. However, the transmembrane class I HLA-G antigen, G, which is specific to EVTs, is expressed to a very limited extent by VCTs [32]. In order to determine whether the overexpression of X3 and X4 during the first trimester is due to an increase in the proportion of trophoblasts, or other component of the villi, we compared the expression of HLA-G, CK7, and CD34, specific to endothelial cells, and vimentin, specific to mesenchymal cells like fibroblasts during the first trimester and at term [33,34].

The quantity of trophoblasts as shown by the CK7 trophoblast marker at three stages of pregnancy (first trimester and at term) did not show any significant difference. Thus, the upward regulation of X3 and X4 does not seem to be correlated with the total amount of trophoblasts. However, there is a significant difference in the proportion of HLA-G between the first trimester and term, with EVT levels up to 100–1000 times higher in the first trimester. The quantification of vimentin and CD34 confirms that the upregulation of X3 and X4 was not correlated with the quantity of these cell types (mesenchymal and endothelial cells), respectively (Figure 3b). This observation confirms that the increased expression of X3 and X4 during the first trimester is not correlated with the quantity of endothelial cells, fibroblasts, or trophoblasts (VCTs and EVTs) but could probably be due to an adaptation to their environment, notably to oxygen levels.

#### 3.3.2. Overexpression of X3 and X4 in the Male Placenta Correlates with Their Specific Expression in Villous Cytotrophoblasts

Numerous studies show that the sex of the fetus and, by extension, that of the placenta can have an impact on gene expression [35,36,37]. In order to determine whether sex affects the expression of these new exons, and therefore their specific transcript levels, we compared their expression and that of the HLA-G and CK7 genes in samples taken during the first trimester of pregnancy from both female and male placentas.

**Figure 4:**
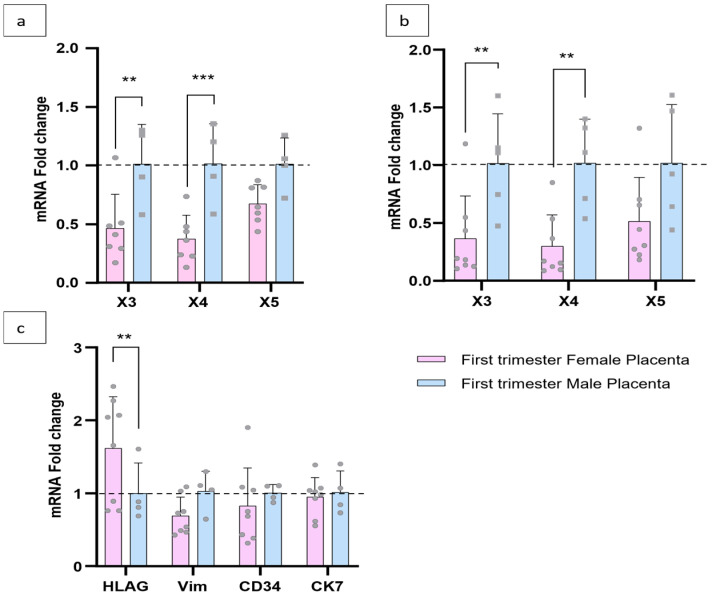
Significant differential expressions of X3 and X4 correlated with the amount of extravillous cytotrophoblasts. (**a**) Relative quantification of X1, X3, X4, and X5, with RPLP0 and SDHA as reference genes, in female and male placentas in the first trimester and at term. (**b**) Relative quantification of X1, X3, X4, and X5, with HLA-G as reference gene, in female and male placentas in the first trimester and at term. (**c**) Relative quantification of HLA-G, vimentin, CD34, and CK7 expression, in female and male placentas, in the first trimester and at term. X1 was barely detectable, and not quantified, **: *p*-value <0.01, ***: *p*-value <0.001. *n* = 7 for the female placenta, and *n* = 5 for the male placenta. Male placenta was used as reference.

We observed a significantly higher proportion of X3 and X4 exons in males than in females, with an equal amount of CK7—a marker of trophoblasts—which means equal amounts of trophoblasts (Figure 4a–c). However, the proportion of HLA-G—a marker of EVT—is twice as high in the female placenta as in the male placenta, and consequently, EVT levels are higher in female than in male villi. These results suggest that only VCTs express X3 and X4. To support this hypothesis, the relative quantification of X1, X3, X4, and X5 with HLA-G as reference gene was carried out and showed overexpression of X3 and X4 in male placentas (Figure 4b). The lower expression of X3 and X4 observed in female placentas could be due to a higher amount of EVT in female than in male placentas. This would lead to a dilution of the amount of VCT-specific X3 and X4 sequences. However, X5 appears to be expressed by both types of trophoblasts—EVTs and VCTs—and maintains an equal expression in male and female placentas regardless of the reference gene used, housekeeping gene, or HLA-G. Moreover, the expression of CD34 (endothelial cells) and vimentin (fibroblasts) is similar in female and male placentas, supporting the specific expression of X3 and X4 by the trophoblast (specifically VCTs) and, therefore, of syncytiotrophoblasts during the first trimester (Figure 4c).

## 4. Discussion

For more than 20 years, PPARγ has been described as essential for placental development and pregnancy maintenance [6,8,38]. Its involvement in placental pathology and in various placental cellular processes, such as trophoblast fusion and invasion, has been clearly established [1,7,39]. Understanding the regulation of the *PPARG* gene in this complex tissue, which undergoes major structural changes during the pregnancy, is necessary to determine the role of each PPARG isoform in placental physiology.

In our study, in silico analysis of transcripts referenced in NCBI revealed four new DNA sequences (termed X1, X3, X4, and X5) which had not been previously described as exons. The *PPARG* gene therefore comprises, overall, 14 exons instead of the ten previously stated. Its structure is close to that described by Omi et al. and confirms the existence of exons A1.1 and A1.2 in the human *PPARG* gene [15]. However, we found an exon, X5, located between exons A2 and B. Chen et al. identified two exons, termed exons C and D, at this location after the alignment of the monkey and human *PPARG* gene [16]. However, we were unable to determine which one corresponded to X5. We confirmed the expression of all the new exons identified, at different levels, in mature PPARG mRNA from placental cells. Each new exon is specific to a transcript. X1, X3, and X4 are included in the transcripts encoding the PPARG1 isoform. However, their expression levels in the VCT differ from one another. Thus, X1 is weakly expressed in the VCT, whereas X3 and X4 are strongly expressed, suggesting that the expression of each transcript is regulated according to the cell type and is not correlated with the protein isoform produced. This hypothesis was confirmed by evidence of expression of exons X3 and X4 mainly by VCT and only slightly by EVT or other cells constituting the villi in samples from first-trimester placentas. The expression of X1, and therefore its existence, although confirmed, was low in placental samples. The specific transcript (NM_001354666.3) including X1 may be specific to a different tissue, not studied here, such as adipocytes or podocytes which express PPARG [40,41]. Moreover, the only upregulation of X4 during formation of the ST suggests its involvement in VCT differentiation and a very strong control of the expression of each transcript, not only in terms of cell type specificity but also throughout cell differentiation. It is well known that PPARγ is strongly involved in lipid metabolism and has a crucial role in the exchange of nutrients across the placenta [42]. Moreover, Tarrade et al. showed in 2001 that the syncytiotrophoblast accumulates more lipids than isolated mononuclear VCTs, which suggests that the transcript NM_001374264.2 could be also involved in this metabolism [43].

In the literature, only four *PPARG* promoters have been reported, upstream of exon A1 (A1.1), A2, B, and exon 1 for the promoters named Pγ1, Pγ3, Pγ2, and Pγ4, respectively [18]. Alignment of transcripts included in the NCBI database shows the possible existence of a total of six promoters, four of which have not been previously described, upstream of X1, A1.2, X3, and X4. Each of them is located in a distal enhancer. This observation could explain the fine regulation of transcript expression but needs to be confirmed in vitro. Nevertheless, the results, based exclusively on the analysis of transcripts included in the NCBI database, did not confirm the presence of two other promoters revealed by Fajas et al. in 1998 and Sundvold et al. in 2001, namely promoters Pγ3 and Pγ4 [18,44,45]. Analysis of other databases could validate these two promoters.

PPARγ is involved in major physiological processes in the placenta: the cellular fusion of VCTs into syncytiotrophoblasts and the migration/invasion of EVTs into the maternal uterine wall [39,46,47]. This second process occurs mainly during the first trimester of pregnancy and is accompanied by an increase in oxygen levels in the intervillous space. PPARγ plays a crucial role in the placenta’s response and adaptation to this increase in oxygen level [48]. Our study revealed the rise in expression of X3 and X4 (but not X1 and X5) during this stage, highlighting the potential implication of the transcript NM NM_001374263.2 and NM_001374264.2 (including X3 and X4, respectively) and not NM_001354666.3 (X1) coding for PPARG1 in the response of the oxygen in the placenta. Their involvement in this process could be confirmed by in vitro culture under hypoxic conditions, which was not carried out as part of this study. The absence of increased expression of X1, which codes for a transcript also translated into PPARγ1, supports the hypothesis of fine regulation of the *PPARG* gene and the expression of each transcript under specific conditions. X5 is specific to the transcript NM_001374262.3 coding for the PPARγ3 isoform and its expression seems to be trophoblast-specific in this study. The quantification of its expression did not show any significant difference during syncytialization of VCT or during the first trimester and the oxygenation of the placenta. Moreover, our results suggest that VCT and EVT express this transcript at the same level. This transcript of the PPARG3 isoform does not appear to be involved in the normal physiology of the placenta. However, some studies of other tissues, such as in adipose tissue or kidney, report PPARG isoforms different from PPARG1 and PPARG2 as dominant negative to PPARG1 and PPARG2 in cell differentiation and pathophysiological conditions [13,40,41,49]. Furthermore, there is no longer any doubt about the involvement of PPARG in the development or progression of cancer [2,50,51,52,53,54,55]. We can hypothesize that several PPARγ isoforms or transcripts could have their expression altered in pathological conditions or in cancers. PPARγ1 plays a key role in placental development and controlling placental vascular proliferation [8]. Meister et al. have, in particular, demonstrated a correlation between PPARγ1 activity and expression and histone modification (H3K4me3 and H3K9ac) in samples taken under physiological conditions and from patients with pre-eclampsia [56]. An increase or disruption in the expression of another isoform of PPARγ, other than PPARγ1, such as PPARγ3, could affect the expression of a lot of genes involved in implantation, vascular remodeling of the maternal uterine arteries, or the endocrine function, growth, and development of the placenta.

## 5. Conclusions

In conclusion, our results show that the human *PPARG* gene is made up of fourteen exons. The differential expression of these new exons suggests a high degree of plasticity in the regulation of *PPARG* gene expression and the possibility of specific functions associated with each RNA transcript and protein isoform.

## Figures and Tables

**Table 1:** Number of samples analyzed of each first-trimester period.

	Gestational Age		
Sex	Before 8 Weeks	Between 8 and 10 Weeks	Between 10 and 14 Weeks
Female	2	2	3
Male	1	2	2

**Table 2:** Primers used in this study.

Target	Forward Primer	Reverse Primer	Tm	Expected Size of Amplicon	Location on the *PPARG* Gene	
X1seq	AACTTACAAACGCAAGGAACTCTG	CTGGTGTCGTTTGCTCCTC	60/59	308	4519	4826
X3	CAAAGAAGTGCCCTGCTAGACAC	TCAAATCTGTTTCAGTTCTTTATCC	61/58	89	18,918	19,006
X4	AGAGCTTCTGTAGCTACGAGATTAT	CTGTTTCAACACTACCCCTTCTT	59/59	62	19,126	19,187
X5	GACGCTGATTTATTTAAATCATCTCT	GGTCATTTCTGGGTTTGCATTCAAG	55/61	97	61,957	62,045
X1	AACTTACAAACGCAAGGAACTCTG	AGTGGCTTGCCCTTCACAC	60/60	164	4519	4683
RPLP0	AACATCTCCCCCTTCTCCT	ACTCGTTTGTACCCGTTGAT	58/58	209		
SDHA	TGGGAACAACAGGGCATCTG	CCACCACTGCATCAAATTCATG	60/59	86		
CK7	GGACATCGAGATCGCCACCT	ACCGCCACTGCTACTGCCA	61/63	124		
HLA-G_1234	ATGGAAGCAGTCTTCCCTGC	TCTTTCTCCACAGCACAGCA	60/60	113		
VIM	CAGGAGGAGATGCTTCAGAT	TGAGGTCAGGCTTGGAAAC	60/58	229		
CD34	GACCCTGATTGCACTGGTCA	GGTTCCAGCTCCAGCCTTT	60/60	117		
Chr X-YF_XR	ATTTGTTCTAAGTCGCCATATTCTCT	GAACACACTACTGAGCAAAATGTATA	59/58	488		
Chr X-YF_YR	ATTTGTTCTAAGTCGCCATATTCTCT	CATCTTTACAAGCTTGTAGACACACT	59/60	340		

All were verified by sequencing, and their efficacies reached 98%. X1seq primers allow the amplification of the whole sequence of the X1 exon and were used for the sequencing. X1 primers were used for the RT-qPCR. The other primers were used for the sequencing and the RT-qPCR.

## Data Availability

The data presented in this study are available in NCBI database https://www.ncbi.nlm.nih.gov/datasets/gene/id/5468/products/ on 1 March 2022. The raw data supporting the conclusions of this article will be made available by the authors on request.

## Supplementary Materials

The following supporting information can be downloaded at: https://www.mdpi.com/article/10.3390/cells15070639/s1, Table S1: Localization in the *PPARG* gene of the exon included in the 5′UTR PPARG transcripts obtained by in silico analysis.

## Abbreviations

The following abbreviations are used in this manuscript:

CK7	Cytokeratin 7
DMEM	Dulbecco′s modified Eagle′s medium
EVT	Extravillous cytotrophoblast
FCS	Fetal calf serum
HLA	Human leukocyte antigen
HBSS	Hank’s balanced salt solution
PPARG	Peroxisome proliferator-activated receptor gamma RNA
*PPARG*	Peroxisome proliferator-activated receptor gamma human gene
PPARγ	Peroxisome proliferator-activated receptor gamma protein
RPLP0	Ribosomal protein lateral stalk subunit P0
SDHA	Succinate dehydrogenase complex flavoprotein subunit A
ST	Syncytiotrophoblast
VCT	Villous cytotrophoblast
